# Epidermoid Cyst in the Floor of the Mouth of a 3-Year-Old

**DOI:** 10.1155/2015/172457

**Published:** 2015-01-28

**Authors:** Rossana Pascual Dabán, Eloy García Díez, Beatriz González Navarro, José López-López

**Affiliations:** ^1^School of Dentistry, University of Barcelona, Barcelona, Spain; ^2^Hospital Materno Infantil Sant-Joan de Déu, Barcelona, Spain; ^3^School of Dental Medicine, University of Barcelona, Barcelona, Spain

## Abstract

Epidermoid cysts are a rare entity in the oral cavity and are even less frequent in the floor of the mouth, representing less than 0.01% of all the cases. We present the case of a 3-year-old girl with a growth in the floor of the mouth with 2 months of evolution and without changes since it was discovered by her parents. The lesion was asymptomatic; it did not cause dysphagia, dyspnea, or any other alteration. A CT scan with contrast was done which revealed the location and exact size of the lesion, allowing an intraoral approach for its excision. The histological examination confirmed the clinical speculation of an epidermoid cyst.

## 1. Introduction

Epidermoid cysts are benign cystic malformations that are derived from the ectoderm [1]. They represent between 1.6 and 6.9% of all the cysts of head and neck [2]; however they are exceptionally rare in the floor of the mouth, representing less than 0.01% of all the cases [1, 3]. Their typical locations are testicles and ovaries. In the head and neck region, the most common location is in the lateral third of the eyebrow [4–7]. This is why we rarely find reported cases of these types of cysts in dental journals (Dentistry, Oral Surgery & Medicine).

Histologically and according to Meyer's classification [8], they can be divided into 3 groups: epidermoid, dermoid, and teratoid. The term dermoid, in addition to representing one of the previous groups, is also classically used to include the three categories [4]. They can be classified as congenital or acquired, although there is no clinical or histological difference between the two [4, 6].

Many theories have been proposed to explain their development, suggesting a dysontogenetic or traumatic origin, or even due to anomalies of the thyroglossal cyst [4]. Those of congenital origin could be the result of the entrapment of ectodermal tissue in the midline between the 1st and 2nd pharyngeal arches during fetal development, around the 3rd and 4th weeks of intrauterine life [2, 4, 9]. On the contrary, the acquired ones could be caused by epithelium implanted deep in the tissue due to trauma or during surgery and are often located away from the midline [7, 10]. They could represent a variant of thyroglossal duct cyst in which the ectodermal tissue is predominant [9], and, finally, some authors suggest the possibility that they originate from the tuberculum impar of His, which with each mandibular arch forms the floor of the mouth and the body of the tongue [6].

Anatomically they are often seen in the midline and we can divide them into three groups based on the relation that they maintain with the muscles of the floor of the mouth: sublingual or medial genioglossus, medial geniohyoid or submental, and lateral, although some authors consider the lateral ones to be medial cysts that expanded laterally, and not a separate entity [4]. In the majority of the cases they are found above the mylohyoid muscle but they can herniate and adopt a typical “hourglass” shape [3].

They are usually slow growing and progressive and always tend to be asymptomatic. When they do not cause displacement or elevation of the tongue due to their size, they can cause dysphagia, dyspnea, and/or the development of a “double chin” if they expand to the submental space [1–3].

They are commonly observed during the 2nd and 3rd decades of life [1, 2, 4, 5] and are rare in children [3, 7], although there is a case described in a newborn [11]. The hormonal stimulation during puberty, along with hypersecretion of fatty tissue, could explain the increased incidence in this age group [9].

The majority of authors believe that there is no sex predilection [3], although authors such as Longo et al. [4] in their report of 16 cases found a greater prevalence in men, with a ratio of 3 : 1.

The size is variable, ranging from a few millimetres to a few centimetres, and within the literature there are three reported cases of giant epidermoid cysts, one of 7 cm and two of 13 cm [1, 12, 13].

## 2. Case Report

**Figure 1:**
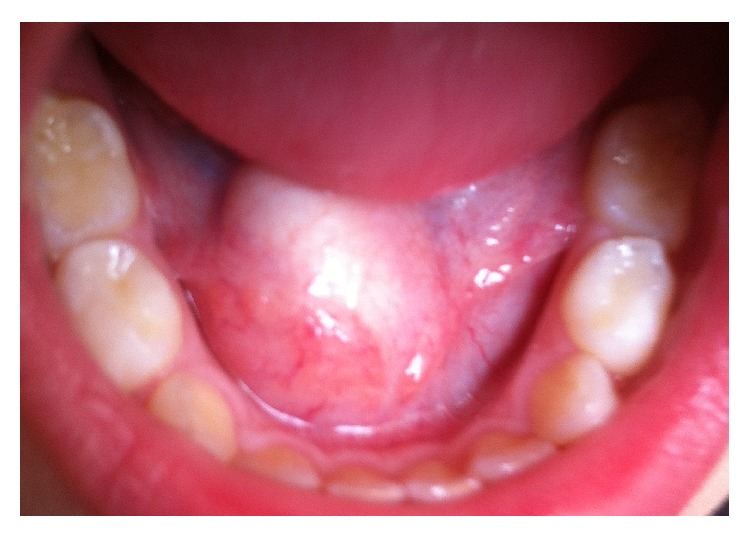
Preoperative photograph of the sublingual mass. An asymptomatic mass can be seen which protrudes discreetly onto the floor of the mouth.

A 3-year-old girl, with no relevant family or personal medical history, presented a growth in the floor of her mouth with two months of evolution, and without any noted changes since it was discovered by her parents. It was asymptomatic and did not cause dysphagia, dyspnea, or any other alteration. There was no palpable lateral cervical lymphadenopathy or fever. On exploration a well-marked growth was observed in the anterior region of the floor of the mouth in the midline. It was covered by normal oral mucosa and did not show signs of inflammation; it was not attached to deeper planes and fluctuated upon palpation (Figure 1).

An initial diagnosis was of a ranula, due to its higher prevalence with respect to the epidermoid cyst, but both its clinical appearance and its location in the midline led us to believe that it could be a dermoid cyst.

**Figure 2:**
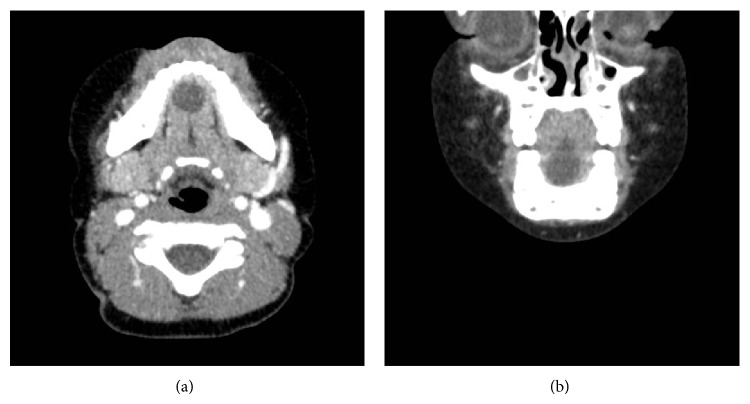
Axial and coronal view of the computerized tomography scan that shows a well-marked cystic mass in the sublingual area.

The CT scan with contrast was done which showed a well-encapsulated cystic mass and confirmed the initial clinical diagnosis (Figures 2(a) and 2(b)).

**Figure 3:**
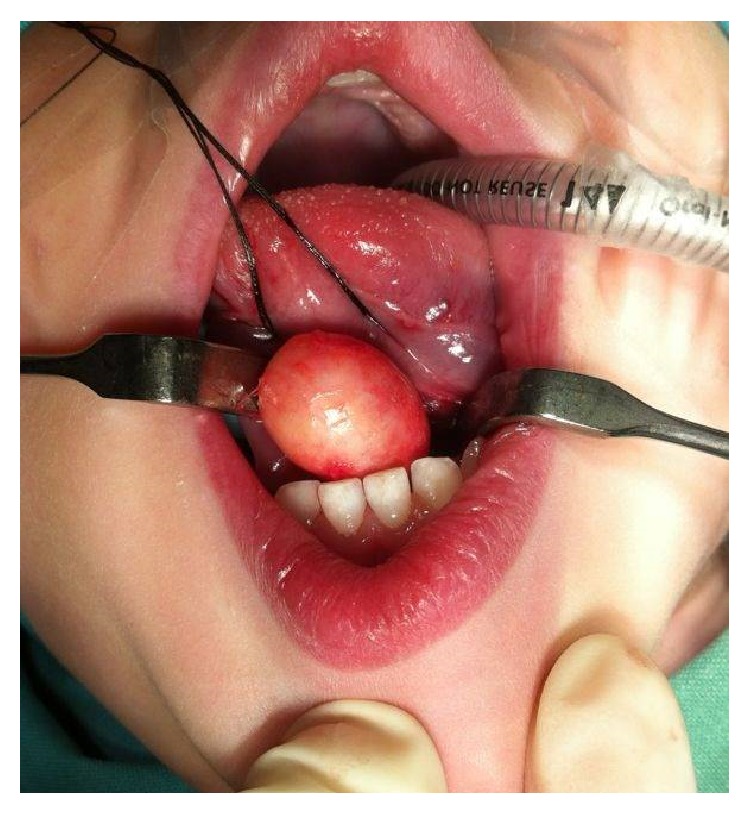
Surgical excision of the lesion.

**Figure 4:**
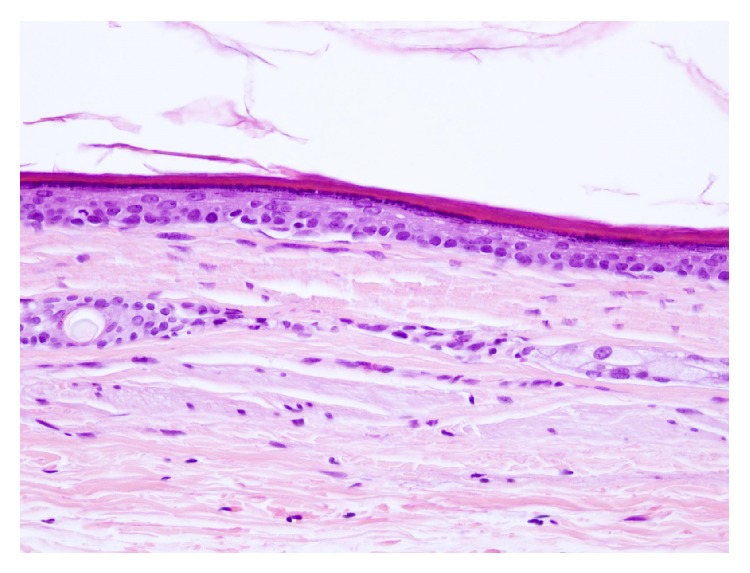
Histopathological examination of the surgical specimen (Haematoxylin and Eosin).

**Figure 5:**
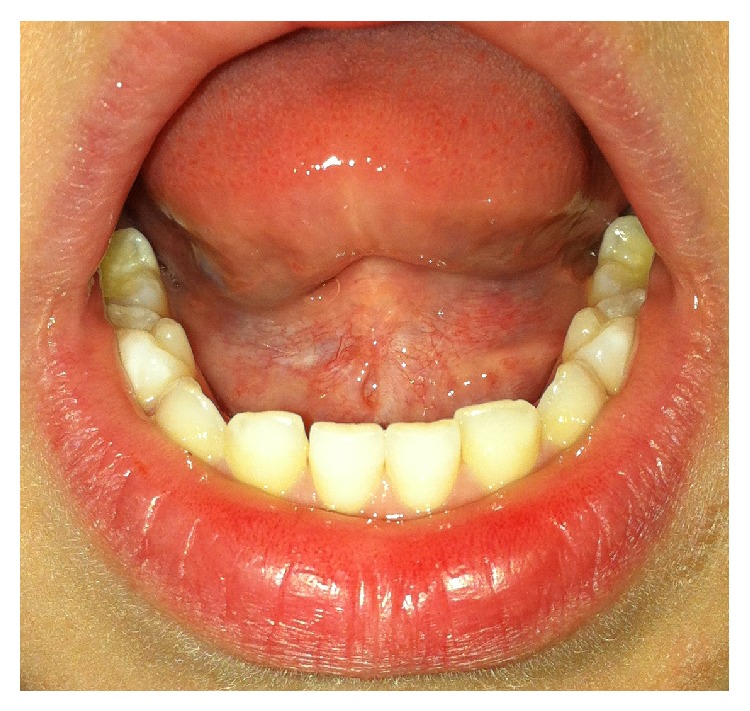
Clinical appearance 6 months after the excision.

The patient underwent surgical treatment of complete excision via intraoral approach. The surgery was performed under general anaesthesia and tracheal intubation which allowed us to make a vertical incision on the ventral aspect of the tongue and blunt dissection was done until the lesion was completely released (Figure 3). The size of the specimen obtained was 2.0 × 1.5 cm which was sent to the Department of Pathology, who then confirmed the diagnosis of an epidermoid cyst (Figure 4). The patient was discharged from the hospital 24 hours after the intervention without any signs of early or delayed complications. No recurrence was observed till a follow-up period of 6 months (Figure 5).

## 3. Conclusion

This pathology is very rare at this location. As cited by Longo et al. [4] in their review of surgical techniques, in 1937 New and Erich already reported 1,495 cases of dermoid cysts and only 24 of them (1.6%) were found in the floor of the mouth. In terms of sex predilection, some authors do not find significant differences, but both in the series of patients found by Longo et al. [4] and in our review of the literature we found a significant predominance in males.

Histologically and in accordance with the classification described by Meyer in 1955 [8], they are divided into three groups: epidermoid cysts which are covered by squamous epithelium that may be partially keratinized and dermoid cysts which also show skin appendages such as hair follicles, hair, sebaceous, and sweat glands. And finally the teratoid variant which also contains elements of the mesoderm, like bone, muscle, and respiratory or gastrointestinal tissue [2]. In our review, as well as in the literature, the epidermoid cysts are the most prevalent group.

The occurrence of complications is not frequent, although they may be infected spontaneously or after an FNAC test (fine needle aspiration cytology) [3]. In our case it was possible to avoid performing an FNAC because both the thorough clinical exploration and the images of the tomography, which could be done without sedation, provided us with sufficient information to suspect a dermoid cyst. However many authors recommend the use of the FNAB [2, 4–6, 9] and they define it as safe, effective, and low-cost measure [2]; some of them describe it as essential [4], although in some cases there was not sufficient information provided in order to make the preoperative diagnosis [9]. Ultrasonography is also suggested as a 1st choice since it is reliable and economical and does not include exposure to radiation [4, 6], but both the CT and MRI are more precise tests with respect to the location and extension of the lesion, allowing the surgeon to choose the best surgical approach [7, 9, 12]. In spite of all these tests, if there are still any doubts, some authors recommend the thyroid scan to rule out ectopic thyroid tissue [6]. Although they are very rare entities, the lingual thyroid or a thyroglossal duct cyst, they are congenital malformations which are more frequently located at the sublingual, suprahyoid, and infrahyoid level [14].

The differential diagnosis must include infectious processes, malignant tumours, extravasation of mucous, and anatomic abnormalities which originate during embryonic development [2, 9]. In our case, we ruled out infection due to the absence of redness, pain, and fever, as well as the time of evolution, and the absence of infectious intraoral areas. We did not believe that we were faced with a malignant tumour because of its clinical appearance and the absence of lymphadenopathy, although we know that the latter is not a precise indicator of malignancy. The lymphatic malformations and lymphohemangioma are considered to be 25% of all benign tumours that affect children; however their location is more typical in the axillary region and head and neck, with the dorsum of the tongue as the main intraoral site. Also, these types of tumours are often present in 65–75% of cases at the time of birth [15].

Since the clinical appearance was compatible with the ranula and these are much more common than epidermoid cysts, it was our first diagnostic hypothesis. The images of the CT, however, made us suspect a dermoid cyst.

Like the rest of the authors, we believe that these growths should be operated on and eliminated in their entirety. This allows for confirming of the diagnosis and avoiding future problems [1–7, 9–13, 16–18]. However, the route of approach is not well established, although it is generally understood that it will depend on the size and location of the lesion. Most authors consider the intraoral route to be the route of choice, and only when it is below the mylohyoid muscle should they consider a submental or mixed approach [2, 3]. In the large cysts initial decompression with aspiration may be necessary since they can pose problems for intubation [1].

If it is completely eliminated, the recurrence of the cyst is rare [1, 2, 4]. Although for the majority of authors there is no possibility of malignant transformation in the epidermoid variant, they consider a teratoma to be the only type that can undergo the transformation [4, 6, 9]. If we review the literature we find an article that reports the appearance of squamous cell carcinoma that arose in the lining of an epidermoid cyst [16].

Finally we can conclude that the dentist should be familiar with the differential diagnosis of the tumour and cystic lesions of the area, in order to be able to perform the appropriate tests for each case and therefore be able to plan a safe surgery.
